# The effect of continuous positive airway pressure on blood pressure in patients with obstructive sleep apnea and uncontrolled hypertension - Study design and challenges during the COVID-19 pandemic

**DOI:** 10.6061/clinics/2021/e2926

**Published:** 2021-08-23

**Authors:** Fernanda C.S.G. Cruz, Luciano F. Drager, Daniel B.C. Queiróz, Gabriela A. Souza, Rodrigo P. Pedrosa, Tarcya L.G Couto Patriota, Egidio L. Dórea, Marcelo Luiz C. Vieira, Camila G. Righi, Denis Martinez, Geruza A. da Silva, Giovanio V. Silva, Andrea Pio-Abreu, Paulo A. Lotufo, Isabela M. Benseãor, Luiz A. Bortolotto, Flávio D. Fuchs, Geraldo Lorenzi-Filho

**Affiliations:** I Laboratorio de Sono, Divisao de Pneumologia, Instituto do Coracao (InCor), Hospital das Clinicas HCFMUSP, Faculdade de Medicina, Universidade de Sao Paulo, Sao Paulo, SP, BR.; II Unidade de Hipertensao, Instituto do Coracao (InCor), Hospital das Clinicas HCFMUSP, Faculdade de Medicina, Universidade de Sao Paulo, Sao Paulo, SP, BR.; III Unidade de Hipertensao, Divisao Renal, Faculdade de Medicina FMUSP, Universidade de Sao Paulo, Sao Paulo, SP, BR.; IV Hospital Universitario, Faculdade de Medicina FMUSP, Universidade de Sao Paulo, Sao Paulo, SP, BR.; V Laboratorio do Sono e Coracao, Pronto-Socorro Cardiologico de Pernambuco (PROCAPE), Universidade de Pernambuco, Recife, PE, BR.; VI Unidade de Ecocardiografia, Instituto do Coracao (InCor), Hospital das Clinicas HCFMUSP, Faculdade de Medicina, Universidade de Sao Paulo, Sao Paulo, SP, BR.; VII Laboratorio Interdisciplinar de Pesquisa em Sono, Hospital de Clinicas de Porto Alegre (LIPES-HCPA), Porto Alegre, RS, BR.; VIII Faculdade de Medicina de Ribeirao Preto, Universidade de Sao Paulo, Ribeirao Preto, SP, BR.; IX Divisao de Cardiologia, Hospital de Clinicas de Porto Alegre, Porto Alegre, RS, BR.

**Keywords:** Obstructive Sleep Apnea, Hypertension, CPAP

## Abstract

**OBJECTIVES:**

To describe the MORPHEOS (Morbidity in patients with uncontrolled HTN and OSA) trial, and describe the challenges imposed by the COVID-19 pandemic.

**METHODS:**

MORPHEOS is a multicenter (n=6) randomized controlled trial designed to evaluate the blood pressure (BP) lowering effects of treatment with continuous positive airway pressure (CPAP) or placebo (nasal strips) for 6 months in adult patients with uncontrolled hypertension (HTN) and moderate-to-severe obstructive sleep apnea (OSA). Patients using at least one antihypertensive medication were included. Uncontrolled HTN was confirmed by at least one abnormal parameter in the 24-hour ABPM and ≥80% medication adherence evaluated by pill counting after the run-in period. OSA was defined by an apnea-hypopnea index ≥15 events/hours. The co-primary endpoints are brachial BP (office and ambulatory BP monitoring, ABPM) and central BP. Secondary outcomes include hypertension-mediated organ damage (HMOD) to heart, aorta, eye, and kidney. We pre-specified several sub-studies from this investigation. Visits occur once a week in the first month and once a month thereafter. The programmed sample size was 176 patients but the pandemic prevented this final target. A post-hoc power analysis will be calculated from the final sample. ClinicalTrials.gov: NCT02270658.

**RESULTS:**

The first 100 patients are predominantly males (n=69), age: 52±10 years, body mass index: 32.7±3.9 kg/m^2^ with frequent co-morbidities.

**CONCLUSIONS:**

The MORPHEOS trial has a unique study design including a run-in period; pill counting, and detailed analysis of hypertension-mediated organ damage in patients with uncontrolled HTN that will allow clarification of the impact of OSA treatment with CPAP.

## INTRODUCTION

Obstructive sleep apnea (OSA) is characterized by recurrent episodes of upper airway obstruction during sleep leading to acute primary responses that are potentially harmful to the cardiovascular system, including excessive negative intrathoracic pressure due to futile effort to breath, sleep fragmentation, and intermittent hypoxia (1). OSA is a common condition in the general population (2) but affects an alarming proportion of patients with hypertension (HTN). It has been estimated that among patients with HTN, 37% have OSA, as defined by an apnea-hypopnea index ≥10 events/hour of sleep (3). Among patients with resistant HTN the prevalence is even higher, and it has been estimated that 64% have moderate to severe OSA (4). The striking prevalence of OSA among patients with HTN is partially explained by an overlap of common risks factors, such as male predominance, obesity, sedentary lifestyle, and advanced age (3). In addition, a body of evidence shows that the primary effects elicited by OSA during sleep trigger a cascade of intermediate responses, such as increased sympathetic activity not only during sleep but throughout the 24-hr period, that contributes to high blood pressure (BP) (5). OSA can also contribute to increase the risk of developing HTN (6), contribute to poor BP control, and to blood vessel and heart remodeling (7).

 The standard treatment of moderate to severe OSA is the application of nasal continuous positive airway pressure (CPAP) during sleep (8). Several investigations have reported a variable but in general modest effect of CPAP on BP. However, a number of limitations of previous studies prevents definitive conclusions (9). Several studies included patients with no HTN, or patients with controlled HTN at study entry, making it difficult to evaluate the impact of OSA treatment on BP due to a floor effect. In addition, several studies had other significant limitations, such as no systematic exclusion of secondary causes of HTN, lack of monitoring antihypertensive adherence, and lack of a placebo arm. As a result, the recent 2017 Guideline for the Prevention, Detection, Evaluation, and Management of High Blood Pressure concluded that the effectiveness of CPAP in BP reduction is not well established in adults with HTN (10). Moreover, the effects of CPAP on central BP have been reported in small studies (11). Central BP provides valuable prognostic information independently of brachial BP (12). It is also worth mentioning that the effects of OSA treatment on hypertension-mediated organ damage (HMOD) deserves additional investigation. Based on the aforementioned gaps in the literature, we planned a randomized controlled trial (RCT) with a unique study design that will help to address several of these issues. This paper intends to report the rationale and design of the MORPHEOS study providing the baseline characteristics of the first 100 patients included in this RCT. We report the challenges imposed by the COVID-19 pandemic and the executive decision to perform a post-hoc power calculation analysis using real data from the final sample.

## METHODS

### Study design

This is an ongoing multicenter, randomized, open-label RCT study designed to determine the effects of OSA treatment with CPAP or nasal dilator (control) for 6 months on BP and several cardiovascular parameters in patients with uncontrolled HTN (Clinical trials.gov: NCT02270658). The institutional ethics committee approved this study (SDC 3230/08/146). Overnight use of a nasal strip is well accepted by patients, and improves subjective daytime sleepiness and depressive symptoms without significant effects in OSA severity (13).

**Figure 1:**
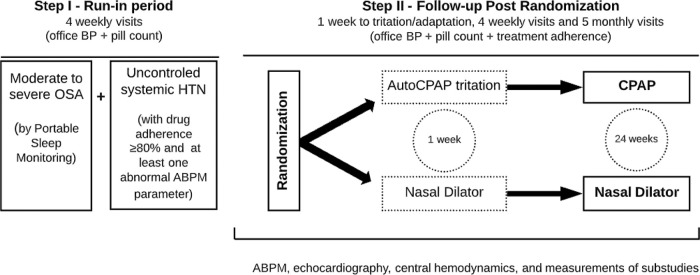
Flow chart.

Consecutive patients with established HTN and clinical suspicion of OSA were recruited from the outpatient clinics of 6 participating sites. Patients with secondary causes of HTN other than OSA were excluded (14). The inclusion and exclusion criteria are described on Table 1. In addition, we used stringent criteria for selecting uncontrolled HTN as determined by pill counting and comprehensive analysis of BP in the run-in period (Step 1). After randomization, the study period has 6 months of follow-up as detailed on Figure 1.

The primary aim of the study is to evaluate the impact of treatment of moderate to severe OSA with CPAP on office BP, 24-hour ambulatory BP monitoring (ABPM), and central BP (co-primary endpoints). Secondary outcomes include markers of HMOD (described below). In addition, a validation study investigating the performance of portable monitoring for the diagnosis of OSA will be performed.

Our hypotheses are that OSA treatment with CPAP will promote a significant decrease in central BP, office BP, and ABPM. In addition, appropriate OSA treatment may decrease left ventricular mass index, improve arterial stiffness, intima-media thickness, and retinal abnormalities. We also hypothesized that portable sleep monitoring is a useful screening tool for OSA in patients with uncontrolled HTN. Although largely used for patients with high clinical suspicion for OSA (15), an appropriate validation in unselected patients with HTN is not available.

### Evaluations

At baseline and after 6 months of follow-up, the following procedures are scheduled:

### Questionnaire

Epworth Sleepiness Scale (ESS): To assess subjective excessive daytime sleepiness, we applied ESS to rank the probability of falling asleep (0 to 3) in eight different situations. If the sum of the scores exceeds 10 points, it is considered positive for the presence of excessive daytime sleepiness (16).

### Portable sleep monitoring

All patients perform a portable overnight sleep recording using the validated device Embletta Gold (PDS; Medcare, Reykjavik, Iceland) (15). In order to validate the portable sleep monitoring in this population, 100 consecutive patients at the Instituto do Coração performed simultaneous full polysomnography, as previously described (17). The sleep study is scored as previously described (18). Apnea is defined by a ≥90% decrease in the airflow from the baseline value for ≥10 seconds. We further classified apnea in obstructive or central based on the presence or absence, respectively, of respiratory-related chest wall excursions. Hypopnea is defined by a ≥30% decrease in the airflow from the baseline value that lasted ≥10 seconds and occurred in conjunction with ≥3% oxygen desaturation (18). OSA is defined by an AHI ≥15 events / hour, because mild OSA seems to not have significant cardiovascular consequences (19).

### Central hemodynamics

The pulsatile pressure and pulse wave increment index determination is performed by a noninvasive applanation tonometry of the radial artery, a SphygmoCor^TM^ device (AtCor Medical, Sydney, NSW, Australia). The radial artery pressure waveforms are recorded in the wrist with a pencil-type high-fidelity micromanometer (Millar Instruments, Houston, Texas). The SphygmoCor^TM^ technique has been previously validated (20). The patient is seated and rests for 5 minutes in a quiet room, after the BP is measured over the brachial artery. After the last measurement, radial artery pressure waveforms of the same arm are sampled over 10 seconds. From the pulse wave analysis of the aortic pressure waveform, the following variables are obtained: aortic pressures, aortic augmentation index (AIx), and AIx adjusted for a heart rate of 75 beats per minute (AIx75). Aortic pressure waveform and Alx are calculated by transfer function of the SphygmoCor device. Aortic Alx is calculated as follows: AIx = ΔP/PP, ΔP=P2-P1 (P2: peak systolic pressure, P1: inflection point that indicates the beginning upstroke of the reflected pressure wave) (21). Only high-quality recordings, defined as an in-device quality index of >80% is accepted for analysis. In general, 2-3 measurements are performed to get two measurements with an acceptable quality index (22). A single trained investigator in each center performed the measurement in a blinded way.

### Office BP

The office BP measurement is carried out with digital sphygmomanometers (Omron^TM^ model HEM - 742) using appropriate cuffs. All BP measures are done after 5 minutes of rest in a sitting position verified by at least 2 BP measurements with a one-minute minimum interval. BP differences >5 mmHg between 1st and 2nd measurements require additional measurements, and the average of all measurements is considered in the calculation (23).

### Ambulatory BP Monitoring – ABPM

The 24-hour ABPM is performed using the SpaceLabs device (model 90207, SpaceLabs Medical, Inc, Snoqualmie, WA) as previously described (24). The BP measurements are performed every 10 minutes during the day and every 20 minutes at night. Activity, bedtime, and time on awakening from sleep are recorded by participants in diaries. Participants are instructed to perform their ordinary daily activities and not to move their arms during the ongoing measurement (23). High BP is defined if at least one parameter (systolic and/or diastolic) is present: mean BP ≥130x80 mmHg (24h); ≥135x85 mmHg (daytime); ≥120x70 mmHg (nighttime) (23). A normal BP dip is defined based on BP as a ≥10% but <20% reduction in BP during sleep compared with during the awake period. Extreme dippers are defined as a ≥20% reduction in BP during sleep compared with the awake period. A non-dipping BP is divided into 2 subtypes: (1) reduced dippers, which is defined as a ≥0% but <10% reduction in BP during sleep compared with during the awake period; and (2) reverse dippers (risers), which is defined as a <10% reduction in BP during sleep compared with during the awake period.

### Echocardiogram

Transthoracic echocardiography is performed using the equipment Vivid E95 GE Healthcare, WI, USA, in a standardized way, according to previous recommendations (25). The acquired data are obtained by using bidimensional (2D) echocardiography, pulsed Doppler, continuous wave Doppler, Tissue Doppler, color flow mapping, and 2D speckle tracking strain. The following are analyzed: left ventricle diameters and volumes (Simpson's rule), left ventricle ejection fraction (Simpson's rule), ventricular septum and posterior wall thickness, relative left ventricle (LV) wall thickness, left ventricle mass index, 2D LV sphericity index, 2D speckle tracking LV longitudinal strain, aortic root diameter, left atrium antero-posterior diameter and biplane volume. Concerning the right ventricle (RV), we analyzed right ventricle diameters (4 chamber view: annular diameter, medium chamber and upper inferior diameter, transversal view diameter), tricuspid annular plane systolic excursion (TAPSE), fractional area change (FAC), S wave by Tissue Doppler, and 2D speckle tracking RV longitudinal strain. LV diastolic assessment is performed by pulsed Doppler or Tissue Doppler as appropriate; mitral valve E and A waves, E/A ratio, deceleration time (DT), isovolumic relaxation time (IVRT), E/e' ratio of the LV annulus, from the basal septal and lateral segments (considered mean values), e'/a’ ratio of basal septal and lateral LV segments. Cardiac valves are analyzed following previous recommendations (26). Pulmonary systolic pressure, tricuspid velocity flow, and RV acceleration time also are measured. All the reports are issued by a single evaluator who is blind to the arm that the participant is randomized to.

### Laboratory tests

Laboratory analysis using standard techniques is performed in all participants and includes glycemia, total cholesterol, low-density lipoprotein (LDL) cholesterol, high-density lipoprotein (HDL) cholesterol, triglycerides, uric acid, ultra-sensitive C-reactive protein (CRP), renin, aldosterone, and microalbuminuria (spot urine). In addition, blood samples (4 aliquots of plasma and 4 aliquots of serum) are stored and immediately frozen at -80° C.

### Step I: Run-in period (selecting true uncontrolled HTN and OSA patients)

All initially selected moderate to severe OSA patients are followed for at least one month to confirm the adherence to medication treatment by pill counting. Uncontrolled HTN is defined by 1) use of at least one antihypertensive medication in a habitual dose; 2) confirmation of good adhesion to therapy by pill counting, 3) elevated BP during ABPM is defined by at least one abnormal parameter (systolic and/or diastolic) present at 24h and/or daytime and/or nighttime periods (23). On the initial visit, the patient brings all current medications to receive instructions on correct antihypertensive drug use for one adherence of at least 80% as previously described by others (27). The participant is advised to not discard the empty boxes, but to bring them along with the other tablets (if any) during the medical visits. From the second visit, the adherence of medication treatment is verified through counting and tabulation of the medicines. The worksheet is filled with antihypertensive drug amounts, dosage, and number of days of treatment. This process is repeated throughout the study.

### Visit 1

The inclusion and exclusion criteria are checked, and the patient signs the consent form. The patient with uncontrolled HTN undergoes clinical evaluation, including a medical history, BP measurements, and physical examination. We performed the first pill count and BP measurements. After the clinical evaluation, we schedule the portable sleep monitoring.

### Visit 2 (∼14 days after Visit 1)

Patients who experience AHI ≥15 events/hour in the portable polygraph exam are evaluated on visit 2. The first drug adherence is performed at this visit, which should be ≥80% of the first count two weeks after visit 1. Office BP measurements are done to ensure the permanence of uncontrolled office HTN. The Epworth Sleepiness Scale is applied.

In the first month, hypertensive patients are followed up by the study participant team. Confirming the maintenance of HTN and adherence to the treatment, the patients undergo ABPM. The examination is useful to rule out patients with controlled BP outside the office.

### Visit 3 (7 days after Visit 2)

Around one week after visit 2, the 24-hour ABPM is performed. After confirming the presence of uncontrolled HTN, echocardiography, central hemodynamic measurement, arterial stiffness, intima-media thickness, ophthalmologic evaluation (including retina and optical coherence tomography analyses), blood collection for biochemical and frozen biobank examinations are scheduled.

### Visit 4 - Randomization (7 days after Visit 3)

One week afterward visit 3, office BP measurements and drug adherence are repeated. Once the participant has all requirements and exams, a sequential random number (starting at 001) is defined. The participants are randomly assigned to receive either CPAP therapy (CPAP group) or nasal dilator (control group). Randomization is performed with the use of a minimization procedure to balance the group assignments according to site. The randomization is done with sealed and numbered opaque envelopes, specific to each center. During the protocol, no changes in antihypertensive medications or dosages are allowed after the randomization procedure.

### Groups

#### CPAP

Participants drawn for CPAP receive the S8 AutoSet II (ResMed™ San Diego, CA) device, and the treatment pressure is determined by the home titration. The values of pressures for the analysis range from 4.0 cm H2O to 20.0 cmH2O. We checked the adherence in the first week by using the software ResScan™. The CPAP pressure 95 percentile values is considered for defining the fixed pressure in the CPAP S8 Elite II™. Good adherence is defined by an average use of at least 4 hours/night. The nasal masks are the first preference (nasal Mirage Micro ResMed™ or nasal Wisp Philips Respironics™). In cases where it is not well tolerated, the Quattro Fx mask ResMed™ is indicated. The masks are adapted according to the size of the nose and face of each participant. Adherence to drug treatment and CPAP is verified during all visits.

#### Nasal dilator strip

This group is assigned to the use of nasal dilator strip (Respire Melhor^TM^, GlaxoSmithKline, Rio de Janeiro, Brazil), which is delivered in medium or large sizes. The participants are instructed to sanitize the external nasal surface with soap and water and to dry it. The adhesive part of the nasal dilator is glued according to the manufacturer’ instructions. The nasal dilator allows a similar attention level for the participant compared with the CPAP arm. Similarly to pill count, nasal dilator count is performed. The nasal dilator is supplied to participants in all visits. Nasal dilator has been shown to be a good placebo for studies testing the effects of CPAP (13).

### Step II: Follow-up After Randomization

#### Visit 1 (7 days after Visit 4 of step I)

On this visit, CPAP pressure is fixed as previously validated (28). The CPAP and nasal dilator adherence checks are started. The adherence to antihypertensive medications is continued.

#### Visits 2-5 (7-day interval between visits)

All participants had weekly visits in the first month to check BP values, antihypertensive medications, and CPAP/nasal dilator adherences. If necessary, additional recommendations for appropriate use of all medications are performed.

#### Visits 6-10 (30-day interval between visits)

The monitoring of BP values, antihypertensive adherence, and CPAP or nasal dilator adherence continues monthly during the subsequent 5 months. If necessary, additional recommendations for appropriate use of all medications are performed.

On visit 14, we apply the Epworth questionnaire and the exams ABPM, echocardiography, central hemodynamics measurement, arterial stiffness, intima-media thickness, ophthalmologic evaluation, blood collection for biochemicals, and frozen biobank examinations are repeated.

### End of evaluations

At the end of the study, patients randomized to CPAP kept their equipment for continuing use following our routine. For ethical reasons, patients randomized to nasal strips also received CPAP and an appropriate follow-up in all centers.

### Safety measures

The antihypertensive therapy is kept unchanged throughout the study. However, if a participant reaches persistent BP limits >180x110 mmHg despite regular use of anti-hypertensive medications, he or she will be excluded from the protocol for safety reasons, returning to the routine of the service to perform appropriate adjustments. In addition, patients with symptoms that may be related to uncontrolled BP, such as uncomplicated headache (with no suspicious of concomitant transient ischemic attack, stroke, or related cerebral diseases) will be treated with symptomatic drugs. If symptoms persisted or additional findings will occur, the decision to change medications and exclude patients from the protocol will be evaluated on an individual basis by an independent Safety Committee.

### Statistical analysis

Intention-to-treat analysis will be used to evaluate the co-primary endpoints (peripheral and central BP). The sample size was initially calculated as 126 patients considering an α-error of 0.05 and 80% power to detect a difference of 5mmHg in peripheral and central BP (standard deviation of 10mmHg). We expected poor adherence in a proportion of patients under CPAP therapy; therefore, our adjusted sample size was 176 patients. However, overall difficulties include financial shortages that make it difficult to provide transportation for study participants and we were surprised by the COVID-19 pandemic. Brazil has been highly impacted by the COVID-19 pandemic; thus, we decided to prematurely terminate the study on July 2020 and perform a post hoc power calculation. We used the site-sealed envelope (https://www.sealedenvelope.com/power/continuous-superiority/). Secondary outcomes, per protocol analyses, include limiting the sample to those with good CPAP and nasal strip adherences are also performed. Portable monitor validation will be performed against the gold standard polysomnography evaluating the sensitivity, specificity, positive and negative predictive values, likelihood ratio, intraclass correlation coefficients, kappa statistics and Bland-Altman plot. Baseline data will be analyzed with SPSS 25.0 (IBM Corporation). Descriptive variables are presented as mean±standard deviation (SD) to continuous variables, and n (%) to categorical variables.

## RESULTS

This is an ongoing study. We report here the first 100 randomized patients. Overall, the population comprised obese middle-aged, white male patients with severe OSA and a significant proportion of co-morbidities including dyslipidemia and diabetes mellitus (Table 2).

## DISCUSSION

In the last decades, a significant body of evidence derived from animal models and clinical studies have pointed to OSA as an important BP modulator not only limited to the sleep period. OSA is considered a secondary cause of HTN and one of the most common conditions associated with resistant HTN (4). However, in clinical practice HTN and OSA frequently co-exist making it difficult to isolate the OSA in BP control. The relationship between OSA and HTN have been questioned after a meta-analysis showing modest effects of CPAP on lowering BP (∼2-3 mmHg) (29) and may be higher in patients with resistant HTN (30). Due to the uncertainties generated by variable results, the European Guidelines of Hypertension in 2018 omitted OSA as a formal secondary cause of HTN placing OSA in the “special conditions” section (31). However, several potential reasons may explain the disappointing effects of CPAP on BP, including the heterogeneity of patients studied (normotensive patients, controlled, and uncontrolled patients with HTN), low CPAP compliance, lack of any adherence method for monitoring antihypertensive medication intake during the trial, no systematic exclusion of secondary HTN and the lack of placebo treatment in the control arm (9). In this scenario, the MORPHEOS study was designed to surpass several of these limitations helping to clarify the relative role of treatment of OSA with CPAP on BP in patients with uncontrolled HTN.

In contrast to the majority of previous studies that evaluated peripheral BP, the MORPHEOS trial also evaluates the effects of CPAP therapy on central BP. Consistent evidence has pointed out that central BP is a marker of poor cardiovascular prognosis independent of office BP (32). More importantly, central pulse pressure is a stronger predictive parameter of cardiovascular events than brachial pulse pressure (12). Central (aortic) peak systolic pressure is lower than brachial systolic pressure, whereas mean and bottom diastolic pressures are generally constant across different sites of the arterial tree, bringing lower pulse pressure in the center than in the periphery (33). Moreover, the heart, kidneys, and major arteries supplying the brain are exposed to aortic rather than brachial pressure. Despite all these advantages, the effects of CPAP on central BP are limited. Hoyos et al. (11) performed a small single-center randomized crossover study with 38 patients with severe OSA to receive 8 weeks of CPAP and sham-CPAP with an intervening 1-month in between the 2 treatment periods. The evaluations are conducted during different hours of the day. Patients randomized to CPAP had a reduction in the central systolic BP of -4.1mmHg, and this effect was not influenced by time-of-day. Nevertheless, the participants in this study are either normotensive or had controlled BP at study entry (11). Moreover, to decrease the baseline variability of factors associated with cardiovascular structural changes, we also excluded patients older than 65 years of age and with BMI ≥40 kg/m^2^ (Table 1). In particular, there are frequent technical limitations for assessing transthoracic echocardiography in patients with BMI ≥40 kg/m^2^. Another unique characteristic of the MORPHEOS study is the use of the nasal dilator strip as a placebo. Although sham-CPAP has been largely used as a placebo in studies evaluating the effects of CPAP on several outcomes, sham-CPAP can cause discomfort and frustration by the necessity of wearing a mask that delivers a suboptimal treatment pressure (13). In contrast, the nasal dilator strip is well accepted by the patients. Moreover, we showed that the nasal dilator strip improved subjective daytime sleepiness and depressive symptoms without significant effects in OSA severity (13), and we therefore considered it a better placebo than sham-CPAP.

The MORPHEOS study will allow the development of several sub-studies. Due to the high prevalence of OSA, portable monitoring is progressively more popular for OSA diagnosis among patients with high pretest probability of OSA. However, portable monitors are not specifically validated among patients with HTN who were not referred to sleep laboratories due to suspected OSA. Therefore, the validation of portable monitoring will contribute to facilitate OSA diagnosis in patients with HTN. Moreover, we are intending to explore the predictors of BP response to CPAP therapy. In addition, the impact of OSA treatment with CPAP on HMOD is still limited. The MORPHEOS study evaluates echocardiographic parameters, arterial stiffness, carotid intima-media thickness, and retinopathy, and renal parameters therefore contributing to the current understanding of OSA treatment with CPAP on these markers.

### Limitations

Despite the aforementioned strengths, we anticipated that the MORPHEOS trial has potential limitations. Because we only include patients with uncontrolled BP, the percentage of exclusion during the study period is high. For ethical reasons, only hypertensive patients receiving regular treatment are recruited. This option prevents any conclusions about the effects of OSA treatment in never-treated patients with HTN. However, anti-hypertensive medications were unchanged during the follow-up (except due to safety issues as previously mentioned in the methods section) which may mitigate this limitation. There is no ideal and feasible method for proving adequate use of medications in clinical practice. Pill counting is largely accepted and has been used in several studies (34), but patients may omit information during the follow-up. Another potential limitation is the relative short-term for evaluating some secondary outcomes. Six months of follow-up may not be enough for reversing heart remodeling or other parameters of HMOD. Preliminary evidence suggests that 3 months of CPAP was able to reduce concentric LV hypertrophy patterns (35). This option followed ethical issues for randomizing patients with severe OSA (including sleepy ones) for >6 months. Finally, the COVID-19 outbreak during the recruitment period has imposed a major challenge on the MORPHEOS trial. The entire world was strongly affected by the viral contamination of Sars-CoV-2. The impact of the pandemic on clinical trials is mainly related to the major limitations in the recruitment. Social distancing and isolation measures was and are necessary to contain the spread of the disease. MORPHEOS study has such a design that several evaluations, exams, and visits are necessary. However, due to the need for social distancing, the patients were unable to reach the site during large periods of the pandemic. Moreover, during the flexible periods, the patients were less likely to come to the center because of fear of contamination. Another aspect of the pandemic is the noise represented by patients who are contaminated with COVID-19 during the trial. In order to avoid the inclusion of patients with missing data and the potential impact of patients who had COVID-19, we decided to prematurely terminate the study and to do a post hoc analysis of the study power.

## CONCLUSIONS

In conclusion, we presented the aims, design, and methods of the MORPHEOS study. In addition, we presented the overall characteristics of participants already randomized, demonstrating the feasibility of the study. The findings of this RCT will extend the identification of the effectiveness of CPAP as a means of treating BP and HMOD in patients with uncontrolled HTN and moderate to severe OSA undergoing regular antihypertensive treatment.

### Participating Sites

#### Southeast Region:

- Instituto do Coração (InCor) do Hospital das Clínicas da Faculdade de Medicina da Universidade de São Paulo, São Paulo, Brasil;
- Unidade de Hipertensão da Divisão de Nefrologia do Instituto Central do Hospital das Clínicas da Faculdade de Medicina da Universidade de São Paulo, São Paulo, Brasil;
- Hospital Universitário da Universidade de São Paulo, São Paulo, Brasil.
- Hospital das Clínicas de Ribeirão Preto da Universidade de São Paulo, Ribeirão Preto, Brasil.

#### South Region:

- Hospital de Clínicas de Porto Alegre da Universidade do Rio Grande do Sul.

#### Northeast Region:

- Pronto Socorro Cardiológico Universitário de Pernambuco.

## AUTHOR CONTRIBUTIONS

Cruz FCSG supervised the study in all centers, investigated and supervised the findings of this work, collected data and wrote the manuscript. Drager LF conceived the original idea, designed the study, coordinated the study, referred participants to the study, investigated and supervised the findings of this work and wrote the manuscript. Queiróz DBC coordinated the study and supervised the study in all centers. Souza GA, Patriota TLGC and Righi CG coordinated the local study and collected data. Pedrosa RP and Fuchs FD coordinated the local study and referred participants to the study. Dórea EL referred participants to the study. Vieira MLC performed echocardiography. Martinez D and Silva GA coordinated the local study and referred participants to the study. Silva GV and Pio-Abreu A referred participants to the study. Lotufo PA and Benseñor IM coordinated the local study. Bortolotto LA referred participants to the study and provided equipment. Lorenzi-Filho G (Principal Investigator) conceived the original idea, designed the study, coordinated the study, referred participants to the study, investigated and supervised the findings of this work, and wrote the manuscript. All authors discussed the results and contributed to the final manuscript.

## Figures and Tables

**Table 1:** Inclusion and Exclusion Criteria.

**Inclusion Criteria**
Age >18 years old
Diagnosis of HTN under treatment with at least 1 antihypertensive drug for at least one month
One or more parameters from ABPM above the recommended limits after the running period
AHI >15 events / hour
**Exclusion Criteria**
Age >65 years old
Body Mass Index ≥40 kg/m^2^
Heart failure, previous acute myocardial infarction and previous stroke
LVEF <45% determined by echocardiography
Moderate or severe valvar disease
Systolic BP >180 mmHg or diastolic BP >110 mmHg
Secondary causes of HTN other than OSA
Chronic renal failure with serum creatinine ≥2 mg / dL
Pregnancy
Cocaine, alcohol, amphetamines, and other illicit drug users
Sympathomimetics use (decongestants, anorectics), oral contraceptives, and nonsteroidal anti-inflammatory drugs

Abbreviations: HTN: hypertension; BP: blood pressure, BMI: body mass index, ABPM: ambulatory blood pressure monitoring; AHI: apnea-hypopnea index; LVEF: left ventricular ejection fraction; OSA: obstructive sleep apnea.

**Table 2:** Baseline characteristics.

Variables	Total (n=100)
Male, n (%)	69 (69)
Age (years), mean (SD)	52±10
Race	
White, n (%)	69 (69)
Black, n (%)	18 (18)
Mixed, n (%)	13 (13)
BMI (kg/m^2^), mean (SD)	32.7±3.9
**Comorbidities**	
Smoking, n (%)	10 (10)
Ex Smoking, n (%)	25 (25)
Dyslipidemia, n (%)	45 (45)
Diabetes Mellitus, n (%)	35 (35)
**Sleep Data**	
AHI (events/hour) mean (SD)	44.9±24
Lowest SpO2 (%)	77 (68-81)
**BP Data**	
Office Systolic BP (mmHg), mean (SD)	153±15
Office Diastolic BP (mmHg), mean (SD)	92±14
Heart Rate (bpm), mean (SD)	76 (66-84)

Abbreviations: IR: interquartile range; SD: standard deviation; BMI: body mass index; SpO2: peripheral oxygen saturation; AHI: apnea-hypopnea index BP: blood pressure. The numeric values are expressed as mean (SD) or median (IR).
